# 529 Laser Scanner Size Effect on Treatment Time

**DOI:** 10.1093/jbcr/irae036.164

**Published:** 2024-04-17

**Authors:** Cassidy Muir, Rohit Mittal, Steven A Kahn

**Affiliations:** MUSC, Charleston, SC; MUSC/SJCH, Charleston, SC; MUSC, Charleston, SC; MUSC/SJCH, Charleston, SC; MUSC, Charleston, SC; MUSC/SJCH, Charleston, SC

## Abstract

**Introduction:**

Patients may develop debilitating scars that cause psychological trauma, chronic pain, hypersensitivity, or limit function following burns or other soft tissue trauma. CO2 laser treatment improves the function and appearance of scars and is an integral step in reconstruction. These treatments are performed under sedation or general anesthesia. Decreasing operative time without compromising outcomes potentially reduces cost and anesthesia related complications, while increasing the number of patients treated. This study compares the treatment time per surface area for two CO2 lasers with 10mm and 15mm scanner sizes. The authors hypothesize that the 15mm scanner size laser is faster and treats more square centimeters per second than the 10mm scanner size laser.

**Methods:**

A single institution, retrospective study of prospectively collected data looked at time to perform laser treatment for an area with 15mm or 10mm lasers by two experienced burn surgeons over 7 months. Injury characteristics, total area treated, and time elapsed during treatment were tabulated. Next, this information was used to calculate the speed of treatment in cm²/s, and the median speeds between lasers compared. A subgroup analysis was performed on a subset of patients who were treated with both lasers and served as internal controls. A two-tailed Mann-Whitney U statistical analysis (p< 0.05) was utilized to compare medians of the two cohorts.

**Results:**

There were 18 and 16 patients treated with the 15mm laser and 10mm laser, respectively. The average speed of the 15mm laser was 3.2 cm²/s while the 10mm was 1.2cm²/s (p < 0.00001). Five patients had the same wounds treated with both lasers, with a median speed of 2.39 cm²/s using the 15mm laser and 0.93 cm²/s with the 10 mm (p< 0.01208). The average energy used with the 10mm laser was 144.1mJ vs 190.2mJ for the 15mm laser (p = 0.05614) while the average density was 2.6 and 3.3 respectively (p< 0.00001).

**Conclusions:**

Using the 15mm laser as compared to the 10mm laser resulted in a 2.67-fold faster operative time per cm² treated while there was no significant difference in energy. As one might anticipate, the larger scanner size was faster.

**Applicability of Research to Practice:**

This information offers information to support decision making for hospital systems to create, support, and expand laser-scar programs. Future studies will be focused on determining the efficacy of the two lasers. These will focus on differences in scar outcomes based on objective scar measurements and identify any differences in depth of penetration, energy and density between the two lasers.

